# Antibacterial innovation: parachutes needed

**DOI:** 10.2471/BLT.23.020823

**Published:** 2023-08-01

## Abstract

Pressure is mounting to define and fund the incentives needed to support antibacterial innovation and launch. Gary Humphreys reports.

Anand Anandkumar’s description of developing and launching a novel antibiotic resonates with the pain of lived experience. “It’s like climbing a mountain and then going over a cliff,” says the Chief Executive Officer and co-founder of Bugworks Research Inc, a biotech company based in Bangalore, India.

The mountain that Bugworks is climbing is biotechnological (the company has a broad-spectrum antibiotic known as BWC0977 undergoing a Phase I trial in Australia), but the cliff the company will be going over – assuming the drug gets through the trial phases and receives regulatory approval – is, essentially, commercial.

Damiano de Felice, director of development at the Combating Antibiotic Resistant Bacteria Biopharmaceutical Accelerator (CARB-X), a global non-profit partnership focused on supporting early-stage antibacterial research and development (R&D), explains: “The more antibiotics are used, the more quickly microorganisms develop resistance to them. The public health imperative of antibiotic stewardship necessarily constrains demand.”

Moreover, the antibiotics to be developed happen to be needed – for the most part – in the low- and middle-income countries that carry the bulk of the infectious disease burden imposed by pathogens singled out by the World Health Organization (WHO) and the Centers for Disease Control and Prevention (CDC) in the United States of America (USA) as priority concerns – India being an example.

“It’s like climbing a mountain and then going over a cliff.”

Anand Anandkumar

“The priority unmet need is in countries where there isn’t a developed market for new antibiotics, or where margins are likely to be razor thin,” observes Seamus O’Brien, director of R&D at the Global Antibiotic Research & Development Partnership (GARDP), a non-profit organization supporting the development of antibiotics from late-stage clinical trials to ensuring access to the new drug.

Combined with limited sales revenues, the high cost of antibiotic development (in excess of 1 billion United States dollars (US$) over a 10 to 15 year period if the cost of failures is included), manufacturing and distribution results in a singularly unprofitable business.

These kinds of challenges have, over the past 30 years, driven the largest biotech companies out of antibacterial R&D. Whereas in the 1980s, some 18 large companies were developing antibiotics, today just a handful remain. The result has been a shrinking of the R&D pipeline and an absence of new drugs.

“Most newly approved antibiotics belong to existing classes where resistance mechanisms are established,” explains Alexandra Cameron, an expert working on R&D in WHO’s Antimicrobial Resistance (AMR) Division. “Of the 12 new antibacterial drugs approved between 2017 and 2021, only two – lefamulin and vaborbactam in combination with meropenem – meet one of the four criteria used by WHO to assess innovation.”

Nature’s R&D pipeline, meanwhile, has continued to flow, with multiple pathogenic organisms adapting to the environmental pressures presented by overused and misused antibiotics, including strains of *Klebsiella pneumoniae* and *Escherichia coli*, which have become resistant to nearly all available antibiotics.

The consequences of this innovation imbalance are already being felt. “Antibiotic resistance is estimated to be directly responsible for the deaths of around 1.3 million people per year, while contributing to the deaths of another 5 million people,” says Haileyesus Getahun, director of AMR Global Coordination at WHO. “And the situation is only going to get worse. So, we need new antibiotics employing new mechanisms and modes of action now.”

The responsibility for coming up with those products rests on companies like Bugworks, which now account for over 80% of the investigational antibiotics going through the R&D pipeline.

Whether they can do it is open to question.

Anandkumar is optimistic, but he has his concerns. “People don’t realize just how small the innovation base is,” he says, noting that most biotech companies working in this space have fewer than 50 employees (Bugworks has 32), and many are facing financial challenges.

Companies working on the preclinical agents that are vital to the overall health of the R&D pipeline appear particularly under-resourced. According to a study done by WHO in 2021, just under half of the 103 companies working on preclinical agents had fewer than 10 employees. “These are small and micro-sized companies,” says Cameron. “They tend to struggle financially.”

And then there is the cliff.

Several companies have gone over it. One is California-based biotech company Achaogen, which developed a drug called plazomicin that was approved by the United States Food and Drug Administration in June 2018 for the treatment of complicated urinary tract infections caused by multidrug-resistant *Enterobacteriaceae*. The company filed for bankruptcy in April 2019.

Another is Nabriva Therapeutics, which developed the antibacterial lefamulin in 2006. The drug was approved for the treatment of adults with community-acquired pneumonia in the USA and the European Union in July 2020. Nabriva launched it but couldn’t generate enough sales and announced that it was winding down its business in January of this year.

Several initiatives have been launched to help the innovators, but for the moment they are focused on climbing the mountain.

CARB-X is an example. Set up in 2016, CARB-X has supported 92 R&D projects, 18 of which have entered or completed Phase I clinical trials. Among these, two (both diagnostics) are on the market, seven have secured advanced development partnerships and 12 are in clinical development.

One of these products is now in Phase III trials – Vedanta BioSciences’ VE303, an oral live biotherapeutic product designed to restore gut microbiota to prevent recurrent *Clostridium difficile* infections. The other are all in Phase I trials.

Bugworks’ BWC0977 is among them. CARB-X stepped in with an initial investment of US$2.6 million in 2017, which has since been increased with a cap of US$10.2 million.

De Felice stresses that it is not just about the money. “We also bring a lot of expertise to the table with our company support teams and our portfolio acceleration tools (PAT), which are designed to help multiple product developers address common challenges such as toxicity risks.” The tools can also help developers gain an understanding of how their antibiotic performs against global bacterial strains.

“[This] is a public health failure, and it needs to be addressed urgently.”

Seamus O’Brien

With Bugworks, the PAT data indicated that BWC0977 performed well against multidrug-resistant pathogens sampled worldwide as well as those found in India. “The data confirmed that we were headed in the right direction before progressing into costly clinical trials,” says Anandkumar.

GARDP is also helping with BWC0977, providing support for a cardiac safety study embedded in the ongoing Phase 1 trial. As noted above, the partnership usually focuses on supporting innovators in late-stage clinical trials to launch. At the World Health Summit held in Berlin in October 2019, GARDP unveiled a strategy to deliver five new treatments that address prioritized drug-resistant infections by 2025.

One of GARDP’s candidates is a novel oral treatment for gonorrhoea, known as zoliflodacin, developed by Innoviva Speciality Therapeutics. “The hope is to deliver an oral alternative, to currently recommended injection-based treatment, that also covers drug-resistant infections,” O’Brien explains, noting that the results of a multi-national Phase III trial of the drug, sponsored by GARDP, are expected by the end of the year.

Another prospect is a new antibiotic combination – cefepime-taniborbactam – aimed at treating complicated urinary tract infections, the first Phase III trial of which, supported by GARDP, was successfully completed by Venatorx Pharmaceuticals in 2022.

While such advances are clearly to be welcomed, they do not address the issue of the post-approval cliff. “We still need the parachutes,” says Anandkumar.

What form such parachutes – or ‘pull incentives’ – might take has long been a topic of debate, attention focusing on different ways to ensure that companies have a revenue stream regardless of sales.

Recent efforts to address this question include attempts to calculate the size of the pull required to keep companies interested (and to keep those already committed from going bust). Chief Executive Officer of CARB-X, Kevin Outterson, recently proposed a best estimate of US$ 3.1 billion, the funding to be delivered through some form of subscription mechanism.

Several governments are now considering the introduction of such schemes, led by the United Kingdom of Great Britain and Northern Ireland, where the National Health Service has agreed to pay Pfizer and Shionogi a fixed fee of £10 million (US$ 12.5 million) a year for ten years for two antibiotics.

“While one country providing US$ 12.5 million a year per drug is obviously not enough, a number of countries doing the same, perhaps in line with GDP or some other benchmark, would create a combined pull incentive that could do the trick,” says John Rex, Chief Medical Officer for F2G, an antifungal biotech company, and one of the co-founders of CARB-X.

Getahun believes that more innovative approaches may be required to kick-start urgently needed antibacterial R&D, and suggests that the 2024 United Nations General Assembly would be a good place to galvanize support from Member States.

“We need to ensure that antibiotics are recognized as global public goods, and mobilize global and national resources on a scale commensurate with the challenge faced,” he says.

GARDP’s O’Brien takes a similar view. “People have tended to focus on this as a market failure problem, but it is in fact a public health failure,” he says. “It needs to be addressed urgently.”

**Figure:**
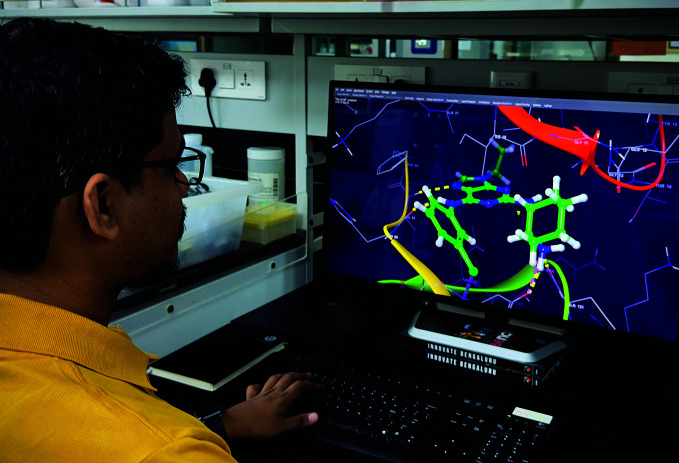
Analysing a digitally modelled microbe as a part of structure-based drug discovery.

Courtesy of Bugworks

**Figure:**
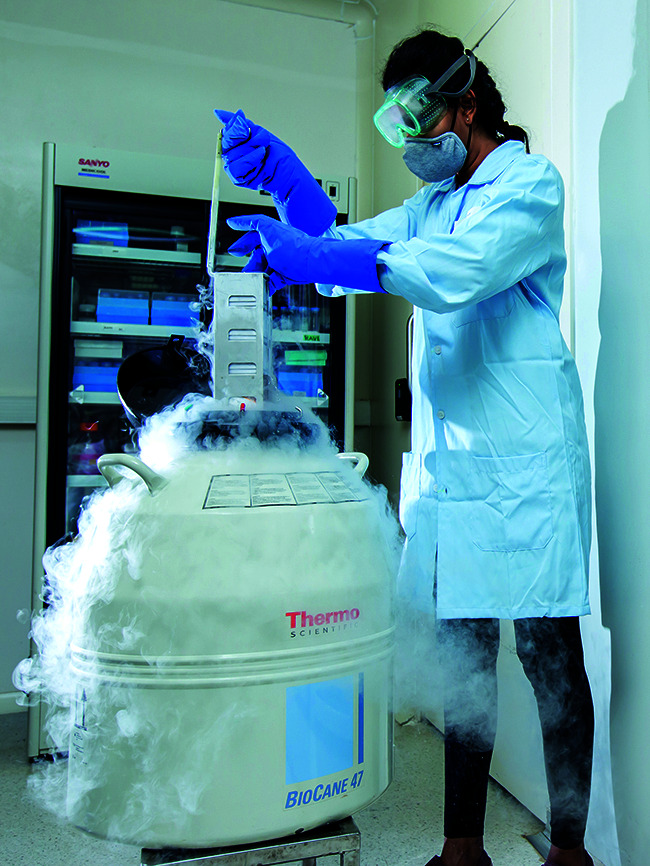
Cryopreserving bacteria for antimicrobial resistance research.

Courtesy of Bugworks

